# Response times are affected by mispredictions in a stochastic game

**DOI:** 10.1038/s41598-024-58203-7

**Published:** 2024-04-10

**Authors:** Paulo Roberto Cabral-Passos, Antonio Galves, Jesus Enrique Garcia, Claudia D. Vargas

**Affiliations:** 1 Departamento de Física da Faculdade de Filosofia, Ciências e Letras de Ribeirão Preto, Universidade de São Paulo, Ribeirão Preto, Brazil; 2 Instituto de Matemática e Estatística, Universidade de São Paulo, São Paulo, Brazil; 3 Instituto de Matemática, Estatística e Computação Científica, Universidade Estadual de Campinas, Campinas, Brazil; 4 Instituto de Biofísica Carlos Chagas Filho, Universidade Federal do Rio de Janeiro, Rio de Janeiro, Brazil

**Keywords:** Learning and memory, Sensorimotor processing, Applied mathematics, Statistics

## Abstract

Acting as a goalkeeper in a video-game, a participant is asked to predict the successive choices of the penalty taker. The sequence of choices of the penalty taker is generated by a stochastic chain with memory of variable length. It has been conjectured that the probability distribution of the response times is a function of the specific sequence of past choices governing the algorithm used by the penalty taker to make his choice at each step. We found empirical evidence that besides this dependence, the distribution of the response times depends also on the success or failure of the previous prediction made by the participant. Moreover, we found statistical evidence that this dependence propagates up to two steps forward after the prediction failure.

## Introduction

More than a century ago, Helmholtz^1^ conjectured that the human brain is able to detect statistical regularities in a sequence of events. Since then, psychophysiological measurements have been employed to study this conjecture^2–11^. Recently, the classical conjecture proposed by Helmholtz was revisited using a new probabilistic framework^12,13^. In Duarte et al.^12^, the relationship between a sequence of auditory stimuli and the sequence of EEG segments recorded during the exposure to these stimuli was modelled using sequences of random objects driven by a stochastic chain with memory of variable length.

Using the framework introduced by Duarte et al.^12^, Hernández et al.^13^ provided statistical evidence that the probability distribution of the EEG segments depended on the smallest sequence of past auditory stimuli governing the choice of the next auditory stimulus. Following Rissanen’s^14^ proposal, the smallest sequence of past stimuli governing the probabilistic choice of the next stimulus is called a *context*. Rissanen also observed that the set of all contexts describing a stochastic chain can be described as the set of leaves of a rooted and labeled tree. For this reason, from now on we will refer to the set of contexts as a *context tree*.

It is natural to conjecture that this dependence on the context tree proposed in Duarte et al^12^ and employed in Hernández et al.^13^ would also occur at a behavioral level. This is the starting point of the present work. Among currently employed behavioral measures, response times have been used to investigate covert processes such as learning of patterns and decision making^2–4,8,15–19^. Modulation of response time has been correlated with the ability to predict a subsequent stimulus^4,20^. Statistical measures extracted from response times^3,4^ suggest that response times emulate the sequence to which they are conditioned to. Besides, errors have been proven to play a role in modulating response times within sequences of events^15–17,19^. In this work we sought to verify the influence of errors on response times in a prediction task in which the sequence of stochastic events is governed by a context tree model. We conjectured that the distribution of response times in a given context would be affected by errors in previous predictions.

To address these issues, we developed a video-game called the Goalkeeper Game^21–23^. In the Goalkeeper game, the penalty taker has three available action choices: kick to the left, to the center, or to the right side of the goal. The sequence of choices of the penalty taker is generated by a stochastic chain with memory of variable length whose dependence on the past is described by a context tree. Acting as a goalkeeper, the participant must predict at each step which will be the next choice of the penalty taker. The participant is instructed to save the maximum number of balls. Response times of the participant are recorded at each trial. After the trial, a feedback video indicates the goalkeeper’s success or failure. The Goalkeeper game offers an opportunity to simulate an environment in which prediction of an upcoming sensorimotor event is necessary and its product is expressed as a prediction success or failure.

In the present framework, we look at the relationship between the probability distribution of response times and the sequence of contexts displayed by the successive choices of the penalty taker. We provide statistical evidence that, besides the dependence on the contexts, the probability distribution of the response times depends also on the success or failure of the previous predictions made by the goalkeeper.

## Methods

### Experimental protocol

**Figure 1:**
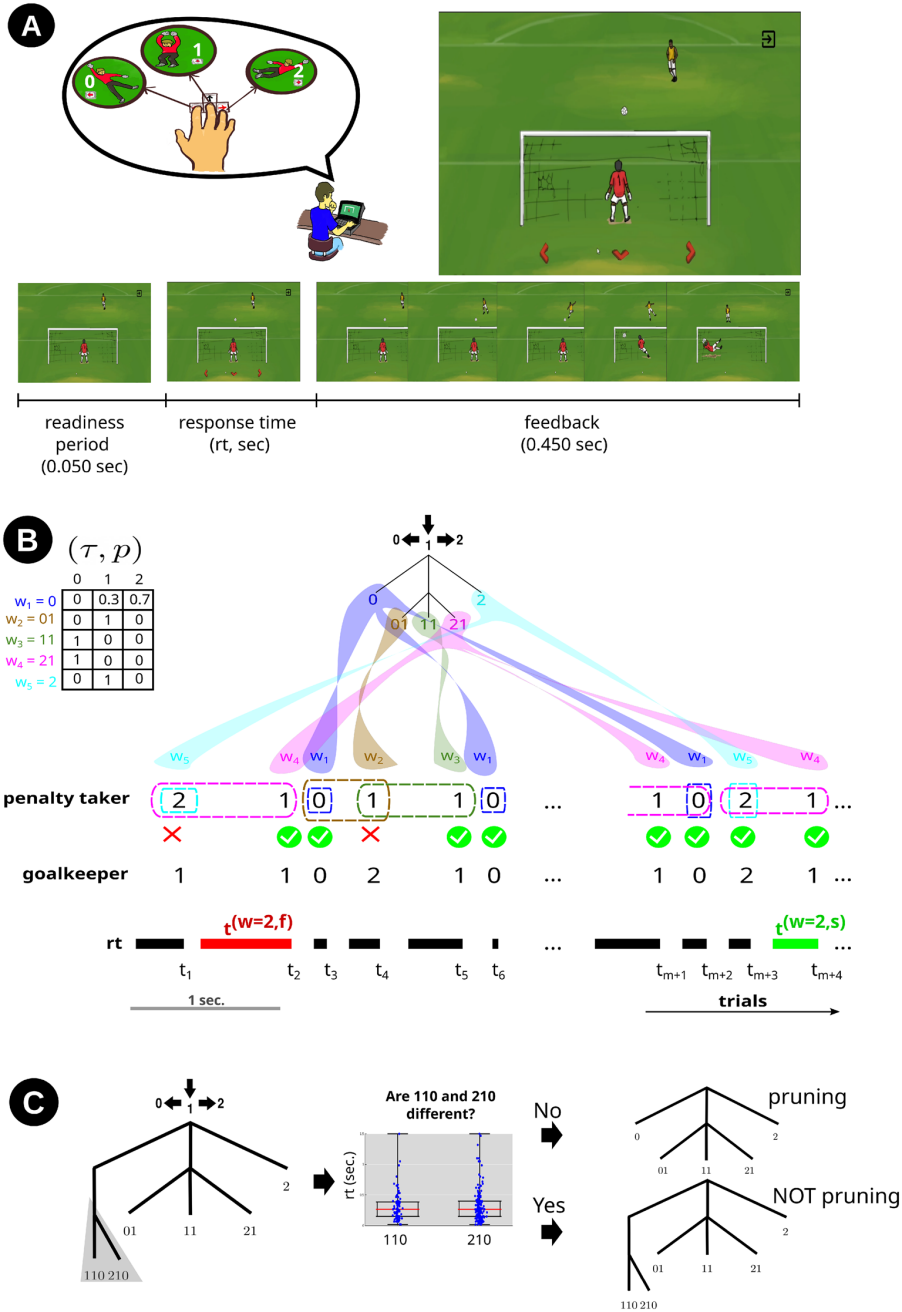
The Goalkeeper Game experiment. (**A**) At each trial, acting as a goalkeeper the participant chooses where to jump to save the kick by pressing the left arrow key with the right index finger (0), the down arrow key with the right middle finger (1) or the right arrow key with the right ring finger (2). The top right picture shows the decision moment of the goalkeeper. The bottom diagram shows the trial’s timeline with the segments duration. Each trial starts with a readiness period. Then, arrows appear at the screen’s bottom, indicating that the participant is allowed to inform his decision by pressing one of the buttons as indicated in the ballons. The time from the appearance of the arrows until the button press is defined as the response time (*rt*). Immediately after the button press, the feedback is presented as an animation depicting the kicker’s choice. (**B**) Top left, the context tree probability table used for generating the penalty taker’s sequence of kicks. On the top right, the set of contexts $\tau$ is represented as a labeled and rooted tree. A different color is attributed to each context. Dashed-line rectangles surround each context in the penalty taker sequence. An example of a penalty taker’s sequence of choices and the corresponding goalkeeper’s sequence of predictions is shown at the bottom. The green check mark indicates a successful prediction, while the red cross ($\times$) indicates a prediction failure. On the bottom, successive *rt*s (*t*) are shown as horizontal bars in which the width represents the *rt* duration in seconds from a real participant. Here, *m* corresponds to a positive integer used to illustrate the shift to trials far from those before the three dots. *Rt*s in the context 2, $t^{(w=2)}$, are highlighted in red after an incorrect prediction, $t^{(w = 2,f)}$, and in green after a correct prediction, $t^{(w = 2,s)}$. (**C**) An example of the pruning procedure used to estimate context trees from *rt*s of a given participant. If the law of *rt*s of at least two leaves of a branch is statistically different, that branch is presented in the estimated tree. The picture illustrates this procedure for the pair of leaves 110 and 210.

The following experiment was performed in accordance with the relevant guidelines and regulations protocol and approved by the Ethics Committee of the Institute of Neurology Deolindo Couto at the Federal University of Rio de Janeiro (CAEE: 58047016.6.1001.5261). All participants had electronically signed their informed consent to participate in the experiment. Twenty-two right-handed participants (14 females) were invited to play remotely the online version of the Goalkeeper Game^21^. In this game version, the participant assumes the role of a goalkeeper in a sequence of 1000 penalty trials. The directions of choice are towards left, center and right. For simplicity, we indicate these directions by the numerical symbols 0, 1 and 2, respectively (Fig. 1A). At each trial, acting as a goalkeeper, the participant chooses where to jump to save the kick by pressing the left arrow key with the right index finger (0), the down arrow key with the right middle finger (1), or the right arrow key with the right ring finger (2). Two rest intervals were placed along the 1000 trials, the first after the trial 334 and the second after the trial 668. The mean and standard deviation of the first and second rest intervals were $54 \pm 55$ s and $50 \pm 40$ s, respectively. The penalty taker choices were not influenced by the previous choices of the goalkeeper. Besides, the goalkeeper was told to take his/her time to make his/her decision and to resume the game after rest intervals. In each trial, the penalty kick took place only after the participant has conveyed his decision by pressing a button.

The sequence of kicks was generated by a stochastic chain with memory of variable length whose dependence on the past is described by a context tree $\tau$. Let *p* be the family of transition probabilities indexed by the contexts in $\tau$, governing the successive choices made by the penalty taker given the corresponding context. The pair $(\tau , p)$ will be called a * probabilistic context tree*^24^.

The probabilistic context tree $(\tau ,p)$ used in our experimental protocol is described in Fig. 1B, which also shows an example of a sequence generated by $(\tau , p)$. This stochastic sequence can also be described as a concatenation of successive choices of the sequence $0 * 1$, where at each repetition the symbol $*$ is replaced either by 2, with probability $p = 0.7$, or by 1 with probability $1{-}p$, independently of the previous choices.

### Analysis

In the following sections, the standard probability theory notation is adopted. In other words, uppercase letters such as *X*, *Y* and *T* are used to indicate random variables and lowercase letters, such as *x*, *y* and *t* indicate the realization of the corresponding random variables.

#### Estimating a context tree from the sequence of response times

Let $(X_{n}: n=1,\ldots , 1000)$ and $(Y_{n}: n=1,\ldots , 1000)$ be, respectively, the sequences of directions chosen by the penalty taker and by the goalkeeper during the game. Both $X_n$ and $Y_n$ belong to the set of possible directions $A=\{0,1,2\}$. We say that the *n*-th prediction is correct when $X_{n} = Y_{n}$. Let also $(T_{n}: n=1,\ldots , 1000)$ be the corresponding sequence of response times of the goalkeeper, see Fig. 1. Given a sequence *w*, *l*(*w*) is the length of *w*.

The following algorithm extends Rissanen’s *Context* algorithm to sequences of real numbers driven by a probabilistic context tree. In the presentation of the algorithm, the word *list* is used in the sense it has in the Python language.

The algorithm uses the *reverse lexicographical order* to arrange the sequences.

##### Definition 1

The reverse lexicographical order between sequences of length *K* is defined as follows: $(u_{-K},\ldots ,u_{-1}) < (v_{-K},\ldots ,v_{-1})$ if either $u_{-1} < v_{-1}$, or there exists $2 \le j \le K$ such that $(u_{-j+1},\ldots ,u_{-1})=v_{-j+1},\ldots ,v_{-1}$ and $u_{-j} < v_{-j}$.

#### Algorithm steps

- **Initialization**: The algorithm begins by initializing an empty context tree ${\hat{\tau }}$ and a list *C*, containing all the sequences of length *K* appearing in the sample.
- **Iterative Process**: The algorithm proceeds in an iterative manner until the set *C* is empty. Within each iteration:

- The first sequence *w* in the list *C* is selected.
- A new list *F*(*w*) is formed. This list contains all the sequences appearing in the sample, that can be obtained by appending, as first element, a symbol from the alphabet *A* to the sequence $(w_{-l(w)+1},\cdots ,w_{-1})$.
- If $F(w)\subseteq C$, the Kolmogorov-Smirnov test is used to decide if the distribution of the response times corresponding to the members of the list *F*(*w*) are the same.
  i. If the Kolmogorov–Smirnov test rejects the equality of distributions, then the sequences in *F*(*w*) are added to ${\hat{\tau }}$ and deleted from the List *C*.
  ii. Otherwise, the sequences in *F*(*w*) are deleted from the list *C* and the sequence $(w_{-l(w)+1},\cdots ,w_{-1})$ is added to the end of *C*.
  iii. In the case of *F(w) = {w}*, *w* is deleted from *C* and $(w_{-l(w)+1},\cdots ,w_{-1})$ is added to the end of *C*.
- If $F(w)\not \subseteq C$, the sequences in $F(w)\cap C$ are deleted from the list *C* and added to ${\hat{\tau }}.$.

1. **Output**: Once all iterations are complete and the list *C* is empty, the algorithm outputs the constructed context tree ${\hat{\tau }}$.

#### Epochs and mode context tree

To access the evolution of the context trees across time, the sequence of response times per participant was divided into three epochs, separated in accordance with the position of rest intervals in the sequence of trials. The first epoch goes from 1 to 334; the second epoch goes from 335 to 668, and the third epoch goes from 669 to 1000. Context trees by epoch and participant were estimated using the algorithm described above. For each epoch, the set of context trees retrieved from the data collected for all the participants was then summarized through a *mode context tree*. The mode context tree contains only the contexts which appear more frequently across participants, see Figure 3 in Hernández et al.^13^ .

#### Response time and accuracy analysis

Given the sequence $w = (w_{-k}, \ldots , w_{-k})$, let $n_{(1,w)}, \ldots , n_{(2,w)}, n_{(3,w)}, \ldots$ be the successive steps ending in the occurrence of *w*. Namely,

$$\begin{aligned} n_{(1,w)}=\min \{n\ge k: ~X_{n-k+1}=w_{-k},\cdots , X_{n}=w_{-1}\}, \end{aligned}$$

and for $$j>1$$

$$\begin{aligned} n_{(j,w)}=\min \{n > n_{(j-1,w)}: ~ X_{n-k+1}=w_{-k},\cdots , X_{n}=w_{-1}\}. \end{aligned}$$

#### Index of correctly predicted transitions

Let $n_{(1,w \rightarrow a)}, \ldots , n_{(2,w \rightarrow a)}, n_{(3,w \rightarrow a)}, \ldots ,$ be the successive steps ending in a occurrence of *w* with correctly predicted transitions from *w* to *a*. That is,

$$\begin{aligned} n_{(j,w \rightarrow a)} = min \{ n \ge n_{(1,w)}:~ X_{n-k+1}=w_{-k},\ldots , X_{n}=w_{-1}\ \text { and } X_{n+1} = Y_{n+1} = a \} \end{aligned}$$

Also, let $N_{ (w \rightarrow a ) }$ be the total number of correctly predicted transitions from *w* to *a*.

Similarly, $n_{(1,w \rightarrow {a}\!\!/ )}, \ldots , n_{(2,w \rightarrow {a}\!\!/)}, n_{(3,w \rightarrow {a}\!\!/)}, \ldots$ be the successive steps ending in a occurrence of *w* with incorrectly predicted transitions from *w* to *a*. That is,

$$\begin{aligned} n_{(j,w \rightarrow {a}\!\!/ )} = min \{ n \ge n_{(1,w)}:~ X_{n-k+1}=w_{-k},\ldots , X_{n}=w_{-1}\ \text { and } X_{n+1} = a \ne Y_{n+1} \} \end{aligned}$$

Also, let $N_{ (w \rightarrow { a }\!\!/ ) }$ be the total number of incorrectly predicted transitions from *w* to *a*.

Given the above notation, we define the index of correctly predicted transitions from *w* to *a* as:

1 $$\begin{aligned} I_{ (w \rightarrow a ) } = \frac{N_{(w \rightarrow a )}}{ N_{(w \rightarrow a )} + N_{(w \rightarrow {a}\!\!/ )} } \end{aligned}$$

#### Response time comparison according to the result of previous predictions

Let $n_{(1,w,f)},n_{(2,w,f)}\ldots$ be the total number successive steps ending in an occurrence of *w* after an incorrect prediction following a 0 in the sequence.

Let *N*(*w*, *f*) be the total number of occurrences of *w* after an incorrect prediction following a 0 and *N*(*w*, *s*) the total number of occurrences of *w* after a correct prediction following a 0.

Let $T_i^{w,s}=T_{n_{(i,w,s)}+1},$ for $i=1,\ldots , N(w,s)$ and $T_i^{w,f}=T_{n_{(i,w,f)}+1},$ for $i=1,\ldots , N(w,f)$ be the set of response times after a correct and an incorrect prediction, respectively, following a 0.

Let ${\bar{T}}^{(w,s)}$ and ${\bar{T}}^{(w,f)}$ be the sample mean response time after a correct prediction and an incorrect prediction following a 0, respectively.

#### Benjamini-Hochberg procedure

To control for false discoveries, the Benjamini-Hochberg procedure was applied whenever multiple comparisons were made^25^. The procedure can be described as follows. Assume that the indexes in parenthesis indicate the ascending order of the corresponding values to which they are associated. Consider $\{H_{m} \}_{m = 1, \ldots , M}$ a set of *M* tested hypothesis such that $p_{m} = p(H_{m})$ are the corresponding *p*-values obtained in each test.

We start by ordering the *p*-values such that:

$$\begin{aligned} p_{(i)} \le p_{ (j) } \text { for all } j > i \end{aligned}$$

Then, for a given false discovery rate *q*, we verify for each *p*-value if

$$\begin{aligned} p_{(i)} \le q \frac{i}{M} \end{aligned}$$

Let *k* be the largest *i* for which the above condition is satisfied. Then, we reject each $H_{(i)}$ for which $i \le k$.

## Results

**Figure 2:**
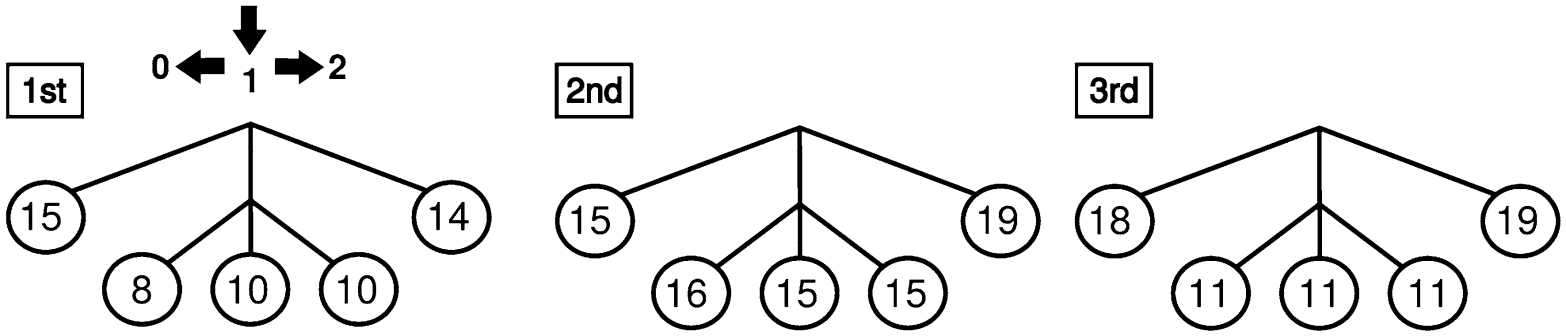
Mode context trees for each epoch. The numbers inside the circles indicate the number of participants (n = 22) for which the corresponding context was present in the estimated trees of that epoch.

Response times were employed to estimate context trees per participant and per epoch (Fig. 2). For all epochs, the mode context tree was the same as the context tree used by the penalty taker to generate the sequence of kicks. Moreover, the number of participants who correctly identified contexts 0 and 2 increased from the first to the third epoch. Curiously, the correct identification of contexts ending in 1 increased from the first to the second epoch, but diminished from the second to the third epoch. Since the sequence of kicks consists in a repetition of $0 * 1$ with $*$ taking the value of 2 with probability $p = 0.7$ and 1 with probability $1-p$, we reasoned that the contexts 01, 11 and 21 might be affected by the congruence between the participants choices and those of the penalty taker.

**Figure 3:**
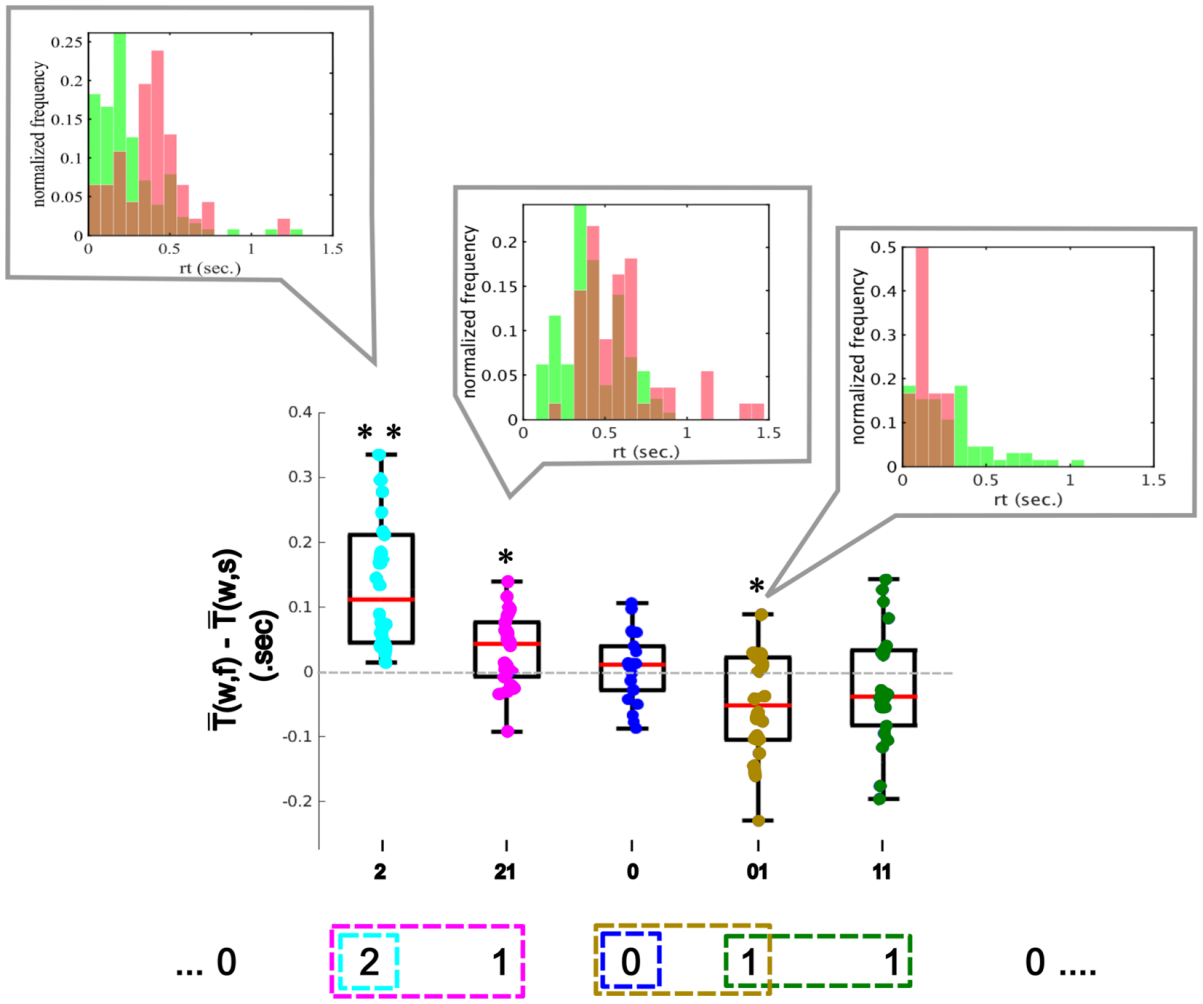
Difference between the mean response time after an incorrect prediction following a 0 ($${\bar{T}}^{(w,f)}$$) and the mean response time after an correct prediction following a 0 ($${\bar{T}}^{(w,s)}$$) for each context. Significance levels of the Wilcoxon signed rank test are indicated by $$*$$ ($$p < 0.05$$) and $$**$$ ($$p = 4.1 \times 10^{-5}$$). All the *p*-values were accepted after applying the Benjamini-Hochberg procedure. Each dot shows the paired difference per participant. On top of each distribution, superimposed histograms of the response times of one participant illustrate the differences between $${\bar{T}}^{(w,s)}$$ in green and $${\bar{T}}^{(w,f)}$$ in red. The sequence of contexts governing the choices of the penalty taker is presented at the bottom.

To evaluate the influence of past predictions over response times in a given context, response times were divided into two sub-samples (see Fig. 1B). $T_1^{(w,s)}, T_2^{(w,s)}, \ldots$ indicate the response times in *w* given that the participant successfully predicted the choice of the penalty taker the last time the context 0 took place. Similarly, $T_1^{(w,f)}, T_2^{(w,f)}, \ldots$ indicate the response times in *w* given that the participant failed to predict the choice of the penalty taker the last time the context 0 took place. This was done because the participant who has learned the regularities of the sequence would only fail to predict the penalty taker’s choice in that context. The mean values of the response times for each participant, context and sub-sample can be found in supplementary table S1.

Figure 3 shows the distributions of response times after correct and incorrect predictions at the last time the context 0 took place, that is, $T_1^{(w,s)}, T_2^{(w,s)}, \ldots$ and $T_1^{(w,f)}, T_2^{(w,f)}, \ldots$, for one participant. To test if the mean values ${\bar{T}}^{(w,f)}$ were significantly different than the mean values ${\bar{T}}^{(w,s)}$, the difference ${\bar{T}}^{(w,f)} - {\bar{T}}^{(w,s)}$ was calculated for each context and participant using the trimmed mean^26^. A two-tailed Wilcoxon signed-rank test showed that these differences were significantly different from zero for the contexts 01, 2 and 21. The test indicated that the mean response times for context $w = 2$ were slower after incorrect predictions compared to after correct predictions ($Z = 4.106$, $p=4.1 \times 10^{-5}$) . This was also true for context 21, which occurs one step further in the sequence, however, with a less pronounced effect ($Z=2.451$, $p = 0.014$). On the other hand, for context $w = 01$, after correct predictions the mean response times were slower than after incorrect predictions ($Z=-0.248$, $p=0.013$). For the context 11 the effect was only close to statistical significance ($Z = -1.379$, $p=0.16$), but it is important to highlight that 11 is the less frequent context of the sequence. Finally, the context 0 presented no significant difference from zero ($Z = 0.478$ ,$p = 0.637$). Taken together, these results indicate that the distribution of response times changes as a function of the result of previous predictions.

**Figure 4:**
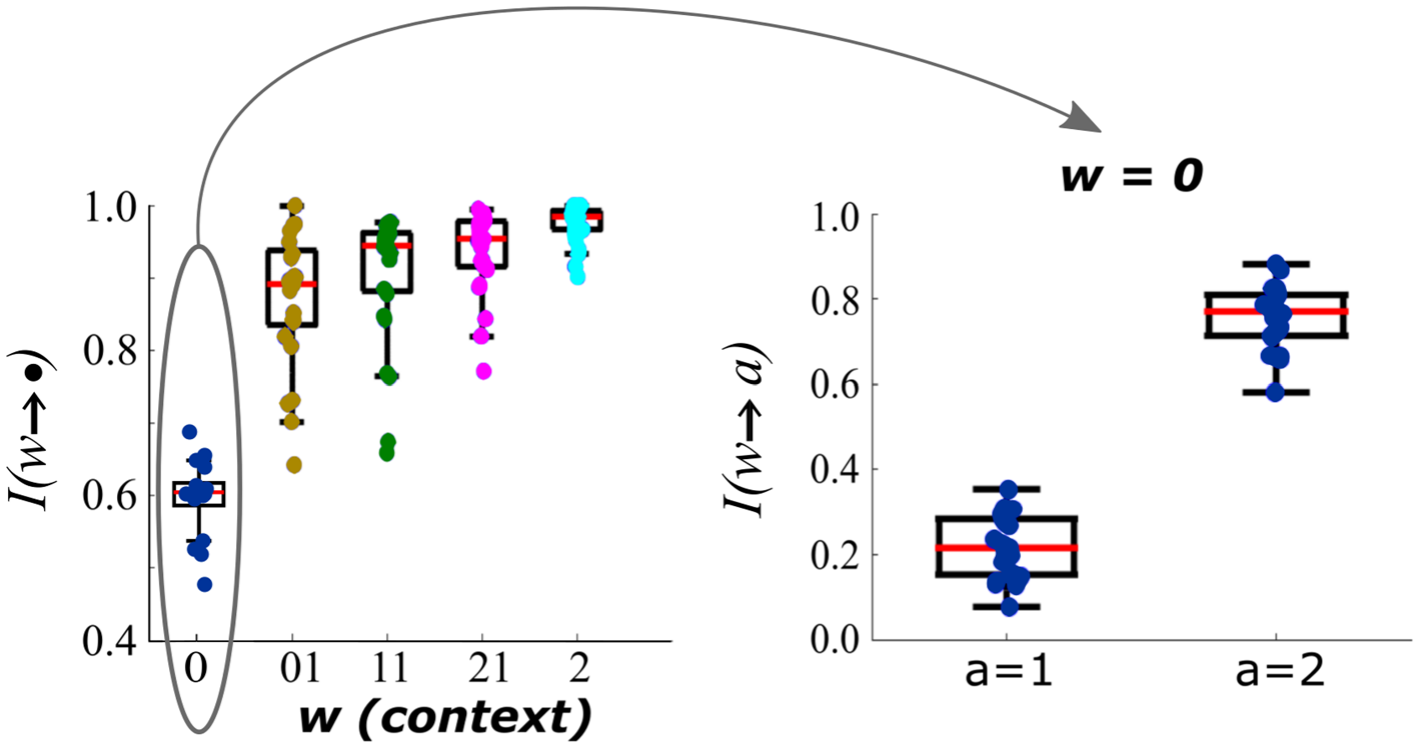
Left: Index of correctly predicted transitions per context. For the non-deterministic context 0 there is more than one transition, as indicated in the right panel. Right: Index of correctly predicted transitions for context 0 given the two possible transitions (1 and 2).

One might conjecture that the response time modulation as a function of contexts shown in Fig. 3 would be related to the relative frequency of correct predictions. If so, for a given context, rare correct predictions would lead to slower response times, whereas frequent correct predictions would lead to faster response times. To evaluate the influence of the proportion of correct versus the proportion of incorrect predictions upon response times, the index of correctly predicted transitions was calculated using Eq. (1) for each context. Figure 4 (left panel) depicts the index of correct predictions per context. It can be noted that this index is high for all the contexts, except for context 0. For this context, the next symbol can be either 1 or 2 (Fig. 4, right panel). When the symbol is 1, the resulting context is 01. For this context, the sample mean of the index of correctly predicted transitions is 0.22. On the other hand, for context 2 the sample mean is 0.75. Taken together, these results indicate that response times are slower after incorrect predictions when compared to correct predictions in the case of a context with a high rate of correct predictions, whereas response times are slower after correct predictions when compared to incorrect predictions in the case of a context with a low rate of correct predictions.

The Kruskal-Wallis test ($\chi ^{2} = 2.55$, $df = 2$, $p = 0.28$) indicated no difference between the response times for different fingers.

## Discussion

Response times associated to a stochastic sequence of events were investigated using the Goalkeeper Game. The sequence of choices of the penalty taker was generated by a stochastic chain with memory of variable length and can be expressed as a repetition of $0~*~1$, in which the middle position $*$ is replaced by a 2 with probability $p=0.7$ and by 1 with probability $1-p$, independently of the goalkeeper’s choices. The statistical analysis of the data provided the following results.

First of all, we successfully retrieved the context tree used by the penalty taker from the goalkeeper’s response time. This supports the conjecture that the probability distributions of the goalkeeper’s response times depend on the contexts governing the choices of the penalty taker at each step. Previous studies reported that response times are affected by the stochasticity of the sequence of stimuli^4,8,10,11^. To the best of our knowledge, this is the first study in which the structure of the sequence of random stimuli is retrieved from the participant’s response times.

Context tree models are mathematical constructs that can efficiently approximate any stationary stochastic chain using a small number of parameters^14,27^, which have been successfully employed to model biological and linguistic phenomena^13,23,27^. A distinct feature of such models is the possibility of straight estimation from a structured sequence of stochastic events. An open question within this framework is whether it is possible to retrieve the context tree model driving the sequence of stochastic events from response times, and most importantly, whether the distribution of response times in a given context would be affected by errors in previous predictions. In the present study, this tool has been employed for the first time to model the unfolding of behavioral responses as a function of sequences of stochastic events driven by context tree models.

We found that the number of participants whose response times allowed to correctly retrieve the penalty taker tree increased from the first to the second epoch of the game. More precisely, in the second epoch, the mode context tree deduced from the response times of a large majority of participants (15 out of 22) coincided with the context tree used to generate the sequence of choices of the penalty taker. Surprisingly, the number of participants whose response times allowed to correctly identify the contexts 01, 11 and 21 decreased from the second to the third epoch (only 11 out of 22 participants). This suggested that an additional factor could be at play. In fact, besides being governed by the context, our statistical analysis provided evidence that response times were also affected by the result of previous predictions.

Response times for a given context depended on the result of previous predictions and this dependence propagated up to two steps forward. This was shown for the contexts 2, 21 and 01, for which different mean response times were identified according to the success or failure of the prediction made by the goalkeeper after the last occurrence of context 0. Slower response times were found after incorrect predictions as compared to correct predictions for the highly predictable contexts 2 and 21. On the other hand, slower response times in the less predictable context 01 were found after correct predictions as compared to incorrect predictions. Several theoretical frameworks were proposed to explore how cognitive control processes affect behavior resulting in the modulation of response times^15,19,28,29^. The cognitive control theory suggests that errors, independently of their frequency, trigger cognitive processes to avoid subsequent errors, resulting in slower upcoming responses^28–30^. The orienting account argues that response time is slower after infrequent events compared to frequent events^15,18,29^. In this case, when errors are frequent, the response time gets slower after correct responses. On the other hand, when errors are infrequent, response times are slower after incorrect responses. That is, the relative frequency of the event triggers the modulation of the response time. In accordance with the orienting account rationale, our results show slower response times occurring after correct predictions in the rarely predicted context 01 and faster response times occurring after correct predictions in the frequently predicted context 2. In conclusion, the modulation of response times in the goalkeeper game is not uniquely associated with making errors, but also depends on the context’s predictability.

One might suppose that the direction of the effect seen in context 01 could be a consequence of inertia, given that there is a repetition of the response with the middle finger (1) in the subsequent trial. On the other hand, switching actions might require deciding between the other two alternatives available, requiring more time to decide, as this calls for a new decision strategy. Comparing response times across fingers revealed no statistical difference, suggesting that the current choice is not significantly affecting the response times in our paradigm.

The Mixed Strategy Nash equilibrium has been extensively employed to model the goalkeeper and kicker’s behaviors in real soccer and other constant-sum games^19,31,32^. The Nash Equilibrium hypothesis states that each player holds the correct expectation about the opponent’s behavior and acts accordingly^33^. In contrast to experimental designs exploring the Nash Equilibrium, in the goalkeeper game the behavior of the penalty taker is completely independent of the goalkeeper’s choice. The goalkeeper, in turn, is affected both by the kicker’s stochastic sequence and by his/her previous choices.

Retrieving the structure of the random sequence of events allows monitoring whether the goalkeeper has learned the law of that stochastic sequence, as indicated by the response time analysis. Furthermore, as the participant learned the context tree model, it was also possible to identify the impact of the prediction outcomes upon response times. This novel approach allowed us to closely inspect the sequence learning process.

In conclusion, we were able to retrieve the statistical regularities from a sequence of response times by applying the Context Tree algorithm^13,23^. This was done by modelling the relationship between the sequence of response times of a given participant and the stochastic sequence of choices of a penalty taker. With this approach, we found that response times are influenced both by contexts and by the results of previous predictions. The Goalkeeper game gives the opportunity to simulate an environment in which prediction is necessary and its product is verifiable. With this information, it is possible to understand new aspects of learning stochastic sequences of sensorimotor events.

### Supplementary Information

Supplementary Table S1.

## Data Availability

The data and the code of the algorithms used in the analysis are available at the following repository: https://github.com/PauloCabral-hub/Publications/tree/main/Passos_etal2023. Instructions about the use of the algorithms are presented in README files included in the repository.
