# Supplementary material for: Response times are affected by mispredictions in a stochastic game
Source: Sci Rep. 2024 Apr 10;14:8446. doi: 10.1038/s41598-024-58203-7 (PMC11006944; doi:10.1038/s41598-024-58203-7)
Supplement: Supplementary file 1 — Supplementary Table S1. [file 41598_2024_58203_MOESM1_ESM.pdf]

Title: RESPONSE TIMES ARE AFFECTED BY MISPREDICTIONS IN A STOCHASTIC GAME
Authors: Paulo Roberto Cabral-Passos, Antonio Galves, Jesus Enrique Garcia, Claudia D. Vargas

Supplementary Material

	$w = 0$		$w = 01$		$w = 11$		$w = 21$		$w = 2$	
ID	*S*	*F*	*S*	*F*	*S*	*F*	*S*	*F*	*S*	*F*
1	0.254	0.265	0.593	0.555	0.256	0.281	0.198	0.212	0.295	0.466
2	0.335	0.347	0.354	0.197	0.445	0.268	0.307	0.286	0.236	0.381
3	0.308	0.413	0.327	0.356	0.354	0.301	0.298	0.346	0.266	0.447
4	0.206	0.267	0.397	0.397	0.177	0.135	0.137	0.224	0.121	0.196
5	0.433	0.365	0.461	0.486	0.396	0.300	0.381	0.351	0.256	0.502
6	0.260	0.258	0.314	0.241	0.235	0.361	0.257	0.321	0.232	0.271
7	0.312	0.321	0.329	0.224	0.255	0.287	0.223	0.263	0.231	0.442
8	0.310	0.349	0.392	0.246	0.247	0.191	0.272	0.246	0.229	0.302
9	0.158	0.171	0.153	0.157	0.141	0.112	0.130	0.095	0.135	0.162
10	0.365	0.462	0.471	0.344	0.330	0.292	0.223	0.284	0.191	0.486
11	0.405	0.362	0.485	0.407	0.435	0.379	0.329	0.374	0.249	0.526
12	0.215	0.276	0.238	0.140	0.247	0.212	0.234	0.226	0.198	0.415
13	0.307	0.319	0.355	0.381	0.446	0.249	0.190	0.306	0.264	0.353
14	0.420	0.342	0.347	0.305	0.249	0.331	0.378	0.436	0.293	0.354
15	0.174	0.236	0.520	0.289	0.151	0.180	0.147	0.149	0.158	0.325
16	0.641	0.590	0.353	0.441	0.380	0.488	0.353	0.452	0.388	0.446
17	0.151	0.160	0.108	0.129	0.120	0.159	0.131	0.132	0.128	0.166
18	0.565	0.477	0.455	0.392	0.401	0.543	0.465	0.372	0.452	0.495
19	0.342	0.373	0.308	0.320	0.393	0.276	0.297	0.436	0.294	0.629
20	0.546	0.532	0.401	0.239	0.380	0.296	0.297	0.373	0.279	0.293
21	0.461	0.432	0.482	0.378	0.427	0.386	0.444	0.540	0.426	0.471
22	0.262	0.233	0.285	0.313	0.304	0.197	0.261	0.271	0.206	0.339

Supplementary Table S1 Mean response times per participant and per context according to the prediction result at the last time the context 0 took place. ID indicates the participant number, w the corresponding context, and S and F, success and failure, respectively.
